# Overcoming phenotypic switching: targeting protein-protein interactions in cancer

**DOI:** 10.37349/etat.2023.00181

**Published:** 2023-10-30

**Authors:** Christos Ladias, Pavlos Papakotoulas, Maria Papaioannou, Nikolaos A. Papanikolaou

**Affiliations:** Istituto Nazionale Tumori-IRCCS-Fondazione G. Pascale, Italy; ^1^Department of Biological Applications and Technology, University of Ioannina, 45110 Ioannina, Epirus, Greece; ^2^First Department of Clinical Oncology, Theageneio Cancer Hospital, 54639 Thessaloniki, Macedonia, Greece; ^3^Laboratory of Biological Chemistry, Department of Medicine, Section of Biological Sciences and Preventive Medicine, Aristotle University of Thessaloniki School of Medicine, 54124 Thessaloniki, Macedonia, Greece

**Keywords:** Protein-protein interactions, phenotypic switching, imatinib, ponatinib, ibrutinib

## Abstract

Alternative protein-protein interactions (PPIs) arising from mutations or post-translational modifications (PTMs), termed phenotypic switching (PS), are critical for the transmission of alternative pathogenic signals and are particularly significant in cancer. In recent years, PPIs have emerged as promising targets for rational drug design, primarily because their high specificity facilitates targeting of disease-related signaling pathways. However, obstacles exist at the molecular level that arise from the properties of the interaction interfaces and the propensity of small molecule drugs to interact with more than one cleft surface. The difficulty in identifying small molecules that act as activators or inhibitors to counteract the biological effects of mutations raises issues that have not been encountered before. For example, small molecules can bind tightly but may not act as drugs or bind to multiple sites (interaction promiscuity). Another reason is the absence of significant clefts on protein surfaces; if a pocket is present, it may be too small, or its geometry may prevent binding. PS, which arises from oncogenic (alternative) signaling, causes drug resistance and forms the basis for the systemic robustness of tumors. In this review, the properties of PPI interfaces relevant to the design and development of targeting drugs are examined. In addition, the interactions between three tyrosine kinase inhibitors (TKIs) employed as drugs are discussed. Finally, potential novel targets of one of these drugs were identified *in silico*.

## Characteristics of protein-protein interaction interfaces

Functional interactions between protein molecules are mediated by protein interfaces, which have specific sizes, shapes, and complementarity characteristics [1, 2] and involve electrostatic and hydrophobic interactions facilitated by the flexibility of the proteins in contact as well as hydrogen bonds. There are three size classes of protein interaction interfaces: The first class is characterized by an average contact area of about 120 to 200 square nanometers, the second by areas of about 110 to 120 square nanometers and occurs in volatile interactions and short-lived complexes [3, 4]. In contrast, proteins involved in signal transduction and protease-substrate interfaces are characterized by larger surfaces, ranging from 200–460 square nanometers. Although the interfaces can be large, as discussed above, few interface residues contribute more than a few kcal/mol of energy (typically > 2 kcal/mol) [5]. Stabilization of protein-protein interactions (PPIs) is determined by the size and chemical character of the interacting surfaces, which are excluded from solvent contacts [6, 7]. The complementarity of the surfaces and the specificity of the interactions are determined by the packing density of the atoms in the two contacting proteins. The forces involved are polar interactions such as hydrogen bonding, electrostatic attractions involving occasional water molecules at the interface, hydrophobic interactions, and van der Waals contacts [8–11]. Protein-protein recognition interfaces and bonds can be identified by measuring the accessible surface area of the interacting proteins, which roughly correspond to the areas of the two proteins that are inaccessible to the water solvent. This restricts the interaction surfaces to clefts or crevices away from water molecules. Stable protein complexes are formed mainly by hydrogen bonds and salt bridges [3], whereas transient complexes are stabilized by hydrophobic interactions [12, 13].

Protein interaction interfaces contain at least three types of atoms: the interface atoms, which lose accessibility to the solvent and are within the van der Waals radii but do not make van der Waals contacts. The second class is the contact atoms, which are all atoms located at a distance corresponding to the van der Waals radii and additionally 0.05 nm away from the atoms of the binding partner. Third, the atoms that are completely buried when two proteins interact, may be accessible to the solvent in the unbound proteins. While the size of the interfaces between different classes of interactions, i.e., antibody-antigen, kinase-substrate, etc., varies considerably, the buried hydrophobic surface area is thought to contribute directly to the binding energy [10, 14]. Notably, hydrophobicity is the main driving force in PPIs, which occur between nonpolar side chains via van der Waals interactions and are thermodynamically spontaneous as the nonpolar domains move from an aqueous to a hydrophobic environment [10, 15]. The hydrophobic effect is the main driving force as the groups of hydrophobic residues withdraw to avoid contact with water, while the H-bonds and electrostatic contacts stabilize the conformation. This leads to the expulsion of water molecules and an increase in entropy, resulting in stable complex formation. Complex formation is also favored by electrostatic attractive forces resulting from the complementarity of the protein interacting surfaces, even within a hydrophobic cleft. Electrostatic interactions determine the half-life of a complex and are typical of transient associations between proteins [16–18].

## Hotspot amino acids and complementarity clusters

Alanine scanning mutagenesis has been extensively used to identify protein interaction sites as well as hotspots, amino acids that provide a large amount of binding energy, typically fixed at 2 kcal/mol [19–21]. The substitution of alanine is advantageous because although it eliminates side groups downstream of the b-carbon, it minimizes the conformational freedom of the protein, which would be increased if glycine were used [22]. Alanine scanning has revealed large differences in the energetic contributions of the replaced interface residues, such that only a few residues participate in the free energy of binding in multiprotein complexes [2, 23, 24]. Typically, it is interaction energies greater than 2 kcal/mol that contribute to the interaction energy in protein pairs. Thus, it is a small number of buried residues that are central to binding affinity, which is determined by calculating the change in binding free energy (ΔΔG_binding_). Alanine scanning revealed that about 10% of the interface residues can be considered hotspots [21, 25]. Interestingly, the same hotspot residues from the same protein are used in different complexes [25–27]. Hotspots are usually conserved residues and are rarely found at the edges of interfaces [5, 28]. The most abundant hotspot amino acids are tryptophan (21%), arginine (13%), and tyrosine (12%). In contrast, leucine, serine, valine, and threonine are almost never used as hotspots in interaction interfaces [20]. Tryptophan is unique in that it can form hydrogen bonds and contributes aromatic π-electrons that form a hydrophobic region and thus can shield hydrogen bonds from nearby competing water molecules. When tryptophan is replaced by alanine in site-directed mutagenesis experiments, the rather large size difference between the two amino acids leaves a significant gap that leads to destabilization. The second most abundant amino acid on hotspots, arginine, can form at least five hydrogen bonds and one salt bridge on the guanidinium motif [20]. Finally, tyrosine, the third most abundant amino acid in hotspots, can interact with its hydrophobic benzene ring and aromatic π-electrons and with the formation of a hydrogen bond from its hydroxyl group. The high percentage of aromatic amino acids as hotspots clearly underlines the importance of the hydrophobic effect in PPIs [29].

## Clustered complementarity of conserved residues

Another feature of protein interaction interfaces is their clustered complementarity of shape and juxtaposed hydrophobic and hydrophilic hotspots. Conserved residues are in pockets throughout the interface and buried charged residues form salt bridges, whereas hydrophobic residues on both surfaces fit well into each other’s cavities and crevices, ensuring complementarity. Thus, complementarity is controlled by the orientation of polar and nonpolar residues, the number of water molecules in the cavities, and the packing density of atoms at the interface [30–32]. Complementary crevices and cavities contain nearly 80% of the hotspot residues and over 90% of the residues with a ΔΔG_binding_ bond greater than 4 kcal/mol are localized in complementary crevices and cavities. Remarkably, these regions contain a small number of polar or ionizable residues, suggesting that the disadvantage of removing water molecules is small, especially since such residues form lamellar bridges within the hydrophobic environment [30].

## Phenotypic switching in protein interactions

Drug resistance is a major challenge in cancer and infectious diseases. Recently, advances in structural biology and chemical reactivity have enabled the design and development of covalent protein interaction inhibitors. A key mechanism of resistance is phenotypic switching (PS) in the formation of protein complexes via alternative PPIs [33]. Drug addiction in cancer cells is also associated with phenotype switching. However, the logic behind the structural and sequence basis of this phenomenon remains currently unclear. Altered interactions in protein complexes underlie almost all phenomena in biology, but they are particularly important in cancer signaling, which maintains tumor growth and metastatic behavior. PS changes in signaling complexes that promote cancer growth arise from mutations [34], post-translational modifications (PTMs), particularly methylation [35, 36] and phosphorylation [37], as well as from other PTMs such as deamidation, ubiquitination and succination [38].

The role of disordered regions is currently being investigated as PS and altered interactions are controlled by conformational changes in the interacting proteins [39, 40]. Altered protein-protein controlled PS is evident in melanoma growth and metastasis and drug resistance [41] and appear to be a general mechanism for tumor growth [42]. However, our understanding of the logic that governs interaction-induced PS in cancers remains limited. This phenomenon is often driven by key mutations that result in conformational changes in constitutively active proteins (e.g., kinases) or the overexpression of oncoproteins (e.g., due to gene amplification). An example of this is drug addiction in melanoma cells which occurs when tumor cells that are initially sensitive to a drug eventually become dependent on its presence for survival [43]. In melanoma cells, the mechanism involves a switch in signaling from the standard v-raf murine sarcoma viral oncogene homolog B1 (BRAF) pathway to the extracellular signal-regulated kinase 2 (ERK2) and JUNB proto-oncogene, AP-1 transcription factor subunit pathways, because when these two genes were genomically knocked out, the melanoma cells died. However, although BRAF signaling can also occur via ERK1, cells remained viable when ERK1 is genomically depleted [44]. The previous argument suggested that the exclusive use of ERK2, as opposed to ERK1, in drug-addicted melanoma cells might result from preferential genetic (or physical) interactions of BRAF complex proteins with ERK2 rather than ERK1 [33]. The structural and sequence basis of this phenomenon is currently unknown. However, identification of the alternative interactions would help, for example, to identify the interacting surfaces between BRAF and ERK2 complexes and to target them with small molecules.

## Targeting PPI interfaces in oncogenic signaling cascades-pitfalls and promise

The robustness of oncogenic properties of cancer cells chiefly derives from altered PPIs. Therefore, systematic mapping of protein interactomes, i.e., total cellular PPIs in cancer cells, is required to identify key interactions and interfaces. In addition, knowledge of protein surface residues at interaction sites can provide insight into how molecular recognition supports tumor development and growth and may aid in the search for rational design of drugs that regulate PPIs or mimic their action [22]. Conformational changes in growth-related proteins, such as occurs in the oncogenic rat sarcoma (RAS)/epidermal growth factor receptor (EGFR)/neurofibromatosis type 1 (NF1) complexes, and altered PPIs, have been implicated in the oncogenic process, allowing them to interact with alternative protein targets and relay their growth signals in an uncontrolled manner. Thus, multiprotein complexes such as protein kinase B (PKB or AKT)-forkhead box O3a (FOXO3a)-14-3-3, murine double minute 2 (MDM2), P53, and Myc-MYC associated factor X (MAX) complexes have been investigated in several studies and have been shown to affect cancer cell growth and death, however how (and why) alternative interactions occur remains unknown [45, 46]. All of the above examples involve altered PPIs as a result of mutations or overexpression of oncoproteins, and they are typical examples of PS [22]. Systematic network assignment of groups of PPIs involved in interaction cancer networks is currently pursued and may shed light on how it occurs [47, 48].

## Challenges in targeting PPIs in cancer

**Figure 1:**
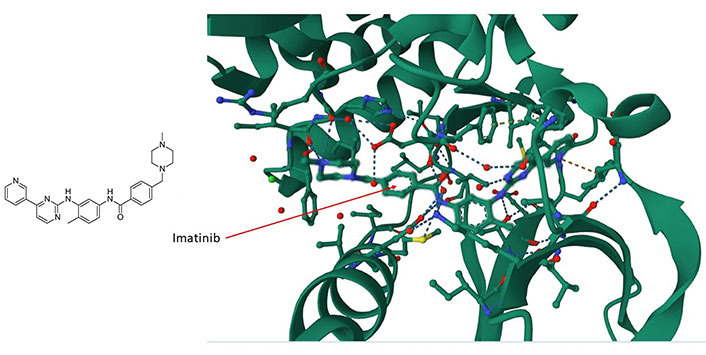
The imatinib/c-ABL complex (PDB code of c-ABL: 6hd6). Imatinib’s chemical structure is shown on the left panel whereas in the right panel, imatinib (shown by the arrow) is situated within a hydrophobic cleft of the c-ABL kinase. It forms interactions with multiple amino acids, including ASP159, HIS139, and PHE160, within a disordered domain. For additional insights, refer to the accompanying text

The large area covered by the PPI interfaces, their noncontiguous nature, the relative lack of natural ligands, and the relative sparseness of deep clefts present formidable obstacles to the implementation of structure-based small molecule design [49, 50]. Nevertheless, there are at least two features that facilitate the development of selective molecular modulators for the future targeting of PPIs. The first feature is the presence of hotspots that contribute most of the binding free energy [51]. The second feature, which counteracts the presence of large interfaces, is the flexibility of side chain motions and unstructured loops on proteins [52, 53]. The presence of disordered regions (about 40% of all proteins are disordered or have extended disordered regions [54]) and the flexibility of atomic motions in proteins allow small molecules to explore their surfaces and bind in ways that cannot always be predicted from static conformational representations [39]. An example is the (promiscuously binding) drug imatinib (Gleevec) which interacts with and inhibits tyrosine kinase c-Abelson leukemia virus protein (c-ABL) (Figure 1), oncoprotein stem cell factor receptor (c-KIT), and the non-kinase NRH: quinone oxidoreductase 2 (NQO2), a flavoprotein that serves as a quinone reductase in the hydroquinone conjugation reaction, in detoxification pathways as well as in biosynthetic, as well as platelet-derived growth factor receptor alpha (PDGFRα) and the Src proto-oncogene, non-receptor tyrosine kinase (SRC) kinase. Imatinib is a tyrosine kinase inhibitor (TKI) that specifically targets the breakpoint cluster region(BCR)-Abelson leukemia virus (ABL) fusion protein in chronic myeloid leukemia (CML) and has beenwidely used in the treatment of various cancers [55]. Imatinib contacts Asp159, His139 and Phe160, among others, in a cleft of c-ABL (Figure 1). Imatinib preferentially inserts itself into a hydrophobic cleft and interacts with a disordered domain (Figure 1), suggesting that the flexibility of these regions favors binding of the drug. The tendency of the drug to preferentially interact with disordered regions in proteins suggests that a broader range of targets remains to be identified and might illuminate the interaction logic of PS mediated by intrinsically disoredered regions (IDRs). The data suggest that similar molecules are likely to bind by a similar mode.

**Figure 2:**
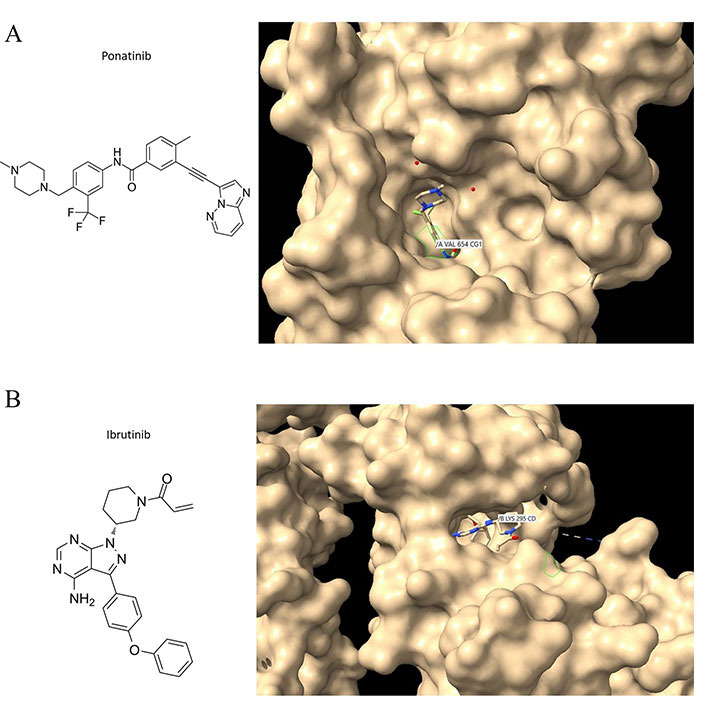
Ponatinib and ibrutinib bound to the c-KIT and c-SRC oncoproteins. (A) C-KIT/ponatinib complex (PDB code:4u0i). The chemical structure of ponatinib (left panel); close aspect of c-KIT with surface features to reveal crevices and pockets and with ponatinib bound to one of these pockets (right panel). VAL654 is in contact with ponatinib; other amino acid contacts include ASP810, CYS809, PHE811, and THR670 (see text for discussion); (B) c-SRC/ibrutinib complex (PDB code:6l8l). The chemical structure of ibrutinib (left panel); Ibrutinib bound to the c-SRC protein showing LYS295 being contacted by the drug (right panel). Interaction analyses were performed with Chimera [56]

Two other similarly structured molecules, ponatinib and ibrutinib, also preferentially bind to deep pockets that contain disordered stretches of amino acids, between secondary structural elements in the targeted kinases (Figure 2A and B) with the tyrosine kinases c-KIT and c-SRC respectively. Ponatinib, a c-ABL1 inhibitor, is currently approved for CML and acute lymphoblastic leukemia (ALL) therapy, and it is also a fibroblast-macrophage colony-stimulating factor (FMSF)-like tyrosine kinase 3 (FLT3) inhibitor. Therefore, the binding characteristics of imatinib can be extrapolated to ponatinib and ibrutinib, as well as other TKIs with similar structures. Regardless, the structural features of a drug and the binding sites on the target protein remain key criteria by which to dissect how a drug interacts with a protein target. It is essential that computational approaches are confirmed with experiment given the propensity of most molecule drugs to bind to several regions and most commonly to hydrophobic crevices or clefts.

Understanding the structural features of the target protein binding sites, facilitates the computational modeling and prediction of the binding affinity and efficacy of similarly structured drugs that target the same or similar protein targets. Imatinib represents an important example of a targeted therapy drug that has revolutionized cancer treatment. However, it is important to emphasize that the binding characteristics of imatinib may not be directly applicable to all other drugs in the same family or to other drugs that target different protein targets [57–59]. A systematic survey of the binding mode of specific classes of drugs will certainly reveal their mode of binding to their targets. Compared to traditional chemotherapy drugs, which often have non-specific effects on rapidly dividing cells, targeted therapy drugs like imatinib can offer more precise and effective treatment with fewer side effects. Regardless, imatinib is not necessarily representative of all targeted therapy drugs, as different drugs can target different proteins with varying levels of selectivity and specificity. Conversely, the ability to bind to multiple proteins within a signaling pathway can be leveraged to achieve broader activity against various types of cancer. Additionally, some targeted therapy drugs may be more, or less, potent than imatinib or may have different pharmacokinetic properties that affect their dosing and administration. Experimental validation is needed to confirm the binding characteristics of other drugs in this family that target different kinases especially because a specific class, the three boron hydrogen (B-H) bonds (BH3)-mimetics, which are designed for breaking PPIs, interact with structured domains of their targets [60, 61] rather than with disordered regions.

**Figure 3:**
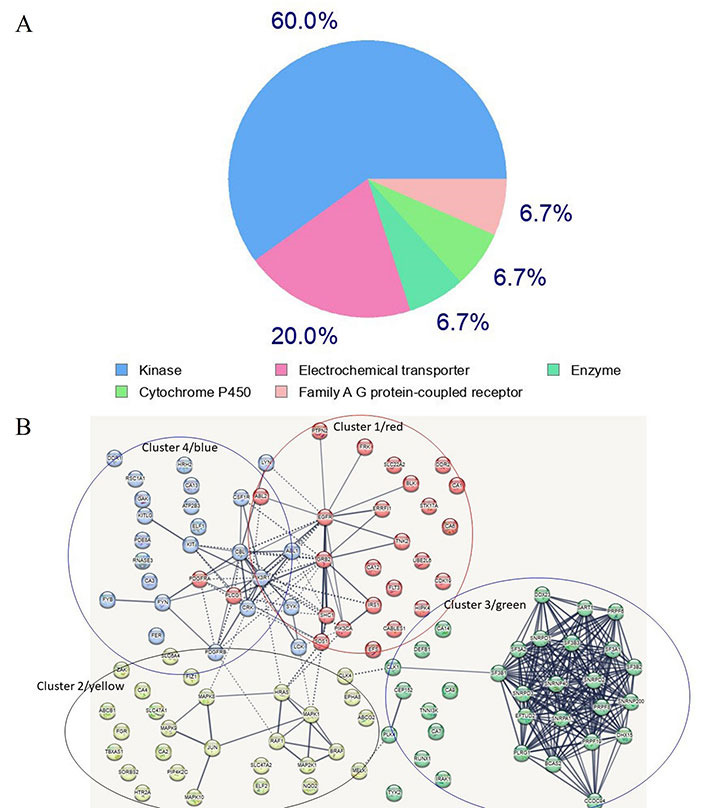
Most frequent *in silico* bound protein classes by imatinib. (A) Gene ontology (GO) analysis of imatinib target proteins. Sixty percent of enriched functional groups are kinase enzymes. A hypergeometric method was used for computing enrichment within the Metascape website [65]; (B) the network of imatinib-targeted proteins that were identified with SwissTarget. Four main clusters are apparent. Clusters 1, 2 and 4 contain well-established enzymes that have been experimentally found to interact with imatinib, whereas cluster 3 contains novel targets identified by *in silico* analysis using the SwissTarget algorithm. The dotted lines in Figure 3B designate the shortest paths between the proteins, with PI3K kinase being a bottleneck for clusters 4 and 1 which share several kinases. Several nodes are unlinked suggesting that not all interactions have been identified or that, if known, were not included in the STRING database

A search for proteins capable of binding imatinib was conducted utilizing the SwissTarget site, resulting in the extraction of several targets (refer to Supplementary material) [62, 63]. Of these targets, 60% are kinases, 20% are electrochemical transporters, and the remainder belong to the family of A/G protein-coupled receptors and other enzymes (Supplementary material, and Figure 3A). A network of the top one hundred computationally-derived targeted proteins was reconstructed utilizing the Search Tool for the Retrieval of Interacting Genes/Proteins (STRING) database. Using K-means clustering, the network was organized [64] into four protein clusters (Supplementary material, and Figure 3B). Not surprisingly, the most enriched proteins in the clusters belong to the signaling kinase receptors (cluster 1), the platelet-derived growth factor receptor beta (PDGFRβ) pathway (cluster 2), the spliceosome and cell division cycle 5-like protein (CDC5L) protein group (cluster 3), and the peptidyl tyrosine phosphorylation and the protein-tyrosine phosphatase 1B (PTP1B) pathways (cluster 4). While clusters 1 and 2 contain proteins known to interact with imatinib, clusters 3 and 4 contain proteins that represent novel, unexplored targets, particularly in the spliceosome and tyrosine phosphorylation pathways (Supplementary material). If experimentally confirmed, it would be interesting to explore whether these disparate protein targets share conformational similarities (especially in the absence of primary structure similarity). Notably, clusters 2 and 4 are linked to cluster 1 whereas cluster 3 appears to be isolated (Supplementary material, and Figure 3B), suggesting that, as of now, not all interactors have been identified. Notably, several kinases in groups 1 (blue) and 4 (red) are clustered together, a finding that is consistent with the targeting preferences of imatinib. A similar analysis for ponatinib and ibrutinib revealed similar targets, however, compared to imatinib’s 60% being kinases, ponatinib and ibrutinib targets overwhelmingly were kinases, 95.3% and 93% respectively (data not shown). Also notably, ponatinib and ibrutinib target novel proteins not targeted by imatinib. Interestingly, phosphoinositide 3-kinase (PI3K) emerged as a top bottleneck in clusters 1 and 4, consistent with its key role in tumors treated with imatinib and other TKIs.

## Conclusions

Understanding the nature of PPIs can be a starting point for developing rational approaches to drug design. Assay technologies that mimic physiology as closely as possible are a necessary step in this direction. In parallel, identifying PPIs driven by rewiring (PS) is critical for discovering systemic drug targets and overcoming drug resistance. A serious obstacle to generalizing rational approaches is the fact that while most small molecule drugs employ the same types of interactions (H-bonds and ionic bridges), it is difficult to predict the site of interaction, as exemplified by the similarly structured drugs, imatinib on the one hand and ponatinib and ibrutinib on the other. In future, modulation of PPIs at the systems level is expected to play an important role in mechanistic studies and in the design and development of systemic drugs.

## Abbreviations

BRAF: v-raf murine sarcoma viral oncogene homolog B1
c-ABL: c-Abelson leukemia virus protein
c-KIT: stem cell factor receptor
ERK2: extracellular signal-regulated kinase 2
PPIs: protein-protein interactions
PS: phenotypic switching
TKIs: tyrosine kinase inhibitors

## Supplementary materials

The supplementary material for this article is available at: https://www.explorationpub.com/uploads/Article/file/1002181_sup_1.xlsx.

## Declarations

### Availability of data and materials

Data are available in the supplementary materials excel file.

### Copyright

© The Author(s) 2023.

## References

[1] Chothia C, Janin J (1975). Principles of protein-protein recognition. Nature.

[2] Lo Conte L, Chothia C, Janin J (1999). The atomic structure of protein-protein recognition sites. J Mol Biol.

[3] Jones S, Thornton JM (1996). Principles of protein-protein interactions. Proc Natl Acad Sci U S A.

[4] Buchwald P (2010). Small-molecule protein-protein interaction inhibitors: therapeutic potential in light of molecular size, chemical space, and ligand binding efficiency considerations. IUBMB Life.

[5] Ofran Y, Rost B (2007). Protein-protein interaction hotspots carved into sequences. PLoS Comput Biol.

[6] Coleman RG, Sharp KA (2010). Protein pockets: inventory, shape, and comparison. J Chem Inf Model.

[7] Levitt M, Park BH (1993). Water: now you see it, now you don’t. Structure.

[8] Persson BA, Jönsson B, Lund M (2009). Enhanced protein steering: cooperative electrostatic and van der Waals forces in antigen-antibody complexes. J Phys Chem B.

[9] Bitencourt-Ferreira G, Veit-Acosta M, de Azevedo WF Jr (2019). Van der Waals potential in protein complexes. Methods Mol Biol.

[10] Xu D, Tsai CJ, Nussinov R (1997). Hydrogen bonds and salt bridges across protein-protein interfaces. Protein Eng.

[11] De S, Krishnadev O, Srinivasan N, Rekha N (2005). Interaction preferences across protein-protein interfaces of obligatory and non-obligatory components are different. BMC Struct Biol.

[12] Bahadur RP, Chakrabarti P, Rodier F, Janin J (2004). A dissection of specific and non-specific protein-protein interfaces. J Mol Biol.

[13] Rodier F, Bahadur RP, Chakrabarti P, Janin J (2005). Hydration of protein-protein interfaces. Proteins.

[14] Tsai CJ, Xu D, Nussinov R (1997). Structural motifs at protein-protein interfaces: protein cores *versus* two-state and three-state model complexes. Protein Sci.

[15] Tang S, Li J, Huang G, Yan L (2021). Predicting protein surface property with its surface hydrophobicity. Protein Pept Lett.

[16] Stevens JM, Armstrong RN, Dirr HW (2000). Electrostatic interactions affecting the active site of class sigma glutathione S-transferase. Biochem J.

[17] Sheinerman FB, Norel R, Honig B (2000). Electrostatic aspects of protein-protein interactions. Curr Opin Struct Biol.

[18] Norel R, Sheinerman F, Petrey D, Honig B (2001). Electrostatic contributions to protein-protein interactions: fast energetic filters for docking and their physical basis. Protein Sci.

[19] DeLano WL, Ultsch MH, de Vos AM, Wells JA (2000). Convergent solutions to binding at a protein-protein interface. Science.

[20] Bogan AA, Thorn KS (1998). Anatomy of hot spots in protein interfaces. J Mol Biol.

[21] Thorn KS, Bogan AA (2001). ASEdb: a database of alanine mutations and their effects on the free energy of binding in protein interactions. Bioinformatics.

[22] Wells JA (1990). Additivity of mutational effects in proteins. Biochemistry.

[23] Wells JA (1991). Systematic mutational analyses of protein-protein interfaces. Methods Enzymol.

[24] Ma B, Wolfson HJ, Nussinov R (2001). Protein functional epitopes: hot spots, dynamics and combinatorial libraries. Curr Opin Struct Biol.

[25] Guney E, Tuncbag N, Keskin O, Gursoy A (2008). HotSprint: database of computational hot spots in protein interfaces. Nucleic Acids Res.

[26] Luque I, Freire E (2000). Structural stability of binding sites: consequences for binding affinity and allosteric effects. Proteins.

[27] Ma B, Shatsky M, Wolfson HJ, Nussinov R (2002). Multiple diverse ligands binding at a single protein site: a matter of pre-existing populations. Protein Sci.

[28] Keskin O, Ma B, Nussinov R (2005). Hot regions in protein–protein interactions: the organization and contribution of structurally conserved hot spot residues. J Mol Biol.

[29] Luitz MP, Zacharias M (2013). Role of tyrosine hot-spot residues at the interface of colicin E9 and immunity protein 9: a comparative free energy simulation study. Proteins.

[30] Li X, Keskin O, Ma B, Nussinov R, Liang J (2004). Protein-protein interactions: hot spots and structurally conserved residues often locate in complemented pockets that pre-organized in the unbound states: implications for docking. J Mol Biol.

[31] Lawrence MC, Colman PM (1993). Shape complementarity at protein/protein interfaces. J Mol Biol.

[32] Maeda MH, Kinoshita K (2009). Development of new indices to evaluate protein-protein interfaces: assembling space volume, assembling space distance, and global shape descriptor. J Mol Graph Model.

[33] Kalosidis NI, Mantsou A, Papanikolaou NA (2018). From driver mutations to driver cancer networks: why we need a new paradigm. Cancer Stud.

[34] Cheng F, Zhao J, Wang Y, Lu W, Liu Z, Zhou Y (2021). Comprehensive characterization of protein-protein interactions perturbed by disease mutations. Nat Genet.

[35] Xu J, Richard S (2021). Cellular pathways influenced by protein arginine methylation: implications for cancer. Mol Cell.

[36] Li B, Kong X, Post H, Raaijmakers L, Peeper DS, Altelaar M (2021). Proteomics and phosphoproteomics profiling of drug-addicted BRAFi-resistant melanoma cells. J Proteome Res.

[37] Qiu Y, Wang Y, Chai Z, Ni D, Li X, Pu J (2021). Targeting RAS phosphorylation in cancer therapy: mechanisms and modulators. Acta Pharm Sin B.

[38] Wang S, Ramamurthy D, Tan J, Liu J, Yip J, Chua A (2020). Post-translational modifications of fumarase regulate its enzyme activity and function in respiration and the DNA damage response. J Mol Biol.

[39] Lin X, Kulkarni P, Bocci F, Schafer NP, Roy S, Tsai MY (2019). Structural and dynamical order of a disordered protein: molecular insights into conformational switching of PAGE4 at the systems level. Biomolecules.

[40] Kulkarni P, Shiraishi T, Kulkarni RV (2013). Cancer: tilting at windmills?. Mol Cancer.

[41] Ramsdale R, Jorissen RN, Li FZ, Al-Obaidi S, Ward T, Sheppard KE (2015). The transcription cofactor c-JUN mediates phenotype switching and BRAF inhibitor resistance in melanoma. Sci Signal.

[42] Kemper K, de Goeje PL, Peeper DS, van Amerongen R (2014). Phenotype switching: tumor cell plasticity as a resistance mechanism and target for therapy. Cancer Res.

[43] Lee RJ, Marais R (2017). Cancer: tumours addicted to drugs are vulnerable. Nature.

[44] Kong X, Kuilman T, Shahrabi A, Boshuizen J, Kemper K, Song JY (2017). Cancer drug addiction is relayed by an ERK2-dependent phenotype switch. Nature.

[45] Hennessy BT, Smith DL, Ram PT, Lu Y, Mills GB (2005). Exploiting the PI3K/AKT pathway for cancer drug discovery. Nat Rev Drug Discov.

[46] Prochownik EV, Vogt PK (2010). Therapeutic targeting of Myc. Genes Cancer.

[47] Hao T, Peng W, Wang Q, Wang B, Sun J (2016). Reconstruction and application of protein–protein interaction network. Int J Mol Sci.

[48] Ivanov AA, Revennaugh B, Rusnak L, Gonzalez-Pecchi V, Mo X, Johns MA (2018). The OncoPPi Portal: an integrative resource to explore and prioritize protein–protein interactions for cancer target discovery. Bioinformatics.

[49] Nussinov R, Tsai CJ, Jang H (2021). Anticancer drug resistance: an update and perspective. Drug Resist Updat.

[50] Liu Z, Zou H, Dang Q, Xu H, Liu L, Zhang Y (2022). Biological and pharmacological roles of m^6^A modifications in cancer drug resistance. Mol Cancer.

[51] Pérot S, Sperandio O, Miteva MA, Camproux AC, Villoutreix BO (2010). Druggable pockets and binding site centric chemical space: a paradigm shift in drug discovery. Drug Discov Today.

[52] Ulucan O, Eyrisch S, Helms V (2012). Druggability of dynamic protein-protein interfaces. Curr Pharm Des.

[53] Eyrisch S, Medina-Franco JL, Helms V (2012). Transient pockets on XIAP-BIR_2_: toward the characterization of putative binding sites of small-molecule XIAP inhibitors. J Mol Model.

[54] van der Lee R, Buljan M, Lang B, Weatheritt RJ, Daughdrill GW, Dunker AK (2014). Classification of intrinsically disordered regions and proteins. Chem Rev.

[55] Vener C, Banzi R, Ambrogi F, Ferrero A, Saglio G, Pravettoni G (2020). First-line imatinib *vs* second- and third-generation TKIs for chronic-phase CML: a systematic review and meta-analysis. Blood Adv.

[56] Pettersen EF, Goddard TD, Huang CC, Meng EC, Couch GS, Croll TI (2021). UCSF ChimeraX: structure visualization for researchers, educators, and developers. Protein Sci.

[57] van Erp NP, Gelderblom H, Guchelaar HJ (2009). Clinical pharmacokinetics of tyrosine kinase inhibitors. Cancer Treat Rev.

[58] Hari SB, Perera BGK, Ranjitkar P, Seeliger MA, Maly DJ (2013). Conformation-selective inhibitors reveal differences in the activation and phosphate-binding loops of the tyrosine kinases Abl and Src. ACS Chem Biol.

[59] Panjarian S, Iacob RE, Chen S, Engen JR, Smithgall TE (2013). Structure and dynamic regulation of Abl kinases. J Biol Chem.

[60] Townsend PA, Kozhevnikova MV, Cexus ONF, Zamyatnin AA, Soond SM (2021). BH3-mimetics: recent developments in cancer therapy. J Exp Clin Cancer Res.

[61] Ivarsson Y, Jemth P (2019). Affinity and specificity of motif-based protein-protein interactions. Curr Opin Struct Biol.

[62] Gfeller D, Michielin O, Zoete V (2013). Shaping the interaction landscape of bioactive molecules. Bioinformatics.

[63] Daina A, Michielin O, Zoete V (2019). SwissTargetPrediction: updated data and new features for efficient prediction of protein targets of small molecules. Nucleic Acids Res.

[64] Szklarczyk D, Kirsch R, Koutrouli M, Nastou K, Mehryary F, Hachilif R (2023). The STRING database in 2023: protein-protein association networks and functional enrichment analyses for any sequenced genome of interest. Nucleic Acids Res.

[65] Zhou Y, Zhou B, Pache L, Chang M, Khodabakhshi AH, Tanaseichuk O (2019). Metascape provides a biologist-oriented resource for the analysis of systems-level datasets. Nat Commun.

